# Enhanced Cytotoxicity of Natural Killer Cells following the Acquisition of Chimeric Antigen Receptors through Trogocytosis

**DOI:** 10.1371/journal.pone.0109352

**Published:** 2014-10-14

**Authors:** Fu-Nan Cho, Tsung-Hsien Chang, Chih-Wen Shu, Ming-Chin Ko, Shuen-Kuei Liao, Kang-Hsi Wu, Ming-Sun Yu, Shyh-Jer Lin, Ying-Chung Hong, Chien-Hsun Chen, Chien-Hui Hung, Yu-Hsiang Chang

**Affiliations:** 1 Department of Obstetrics and Gynecology, Kaohsiung Veterans General Hospital, Kaohsiung, Taiwan; 2 Department of Medical Education and Research, Kaohsiung Veterans General Hospital, Kaohsiung, Taiwan; 3 Department of Pediatrics, Kaohsiung Veterans General Hospital, Kaohsiung, Taiwan; 4 Graduate Institute of Cancer Biology and Drug Discovery and Center of Excellence for Cancer Research, Taipei Medical University, Taipei, Taiwan; 5 Department of Pediatrics, Children's Hospital and School of Chinese Medicine, China Medical University Hospitals, Taichung, Taiwan; 6 Haematology-Oncology Section, Department of Medicine, Kaohsiung Veterans General Hospital, Kaohsiung, Taiwan; 7 Department of Radiation Oncology, Kaohsiung Veterans General Hospital, Kaohsiung, Taiwan; 8 Faculty of Medicine, National Yang-Ming University, Taipei, Taiwan; 9 Department of Nursing, Tajen University, Yanpu Township, Pingtung County, Taiwan; Centre de Recherche Public de la Santé (CRP-Santé), Luxembourg

## Abstract

Natural killer (NK) cells have the capacity to target tumors and are ideal candidates for immunotherapy. Viral vectors have been used to genetically modify *in vitro* expanded NK cells to express chimeric antigen receptors (CARs), which confer cytotoxicity against tumors. However, use of viral transduction methods raises the safety concern of viral integration into the NK cell genome. In this study, we used trogocytosis as a non-viral method to modify NK cells for immunotherapy. A K562 cell line expressing high levels of anti-CD19 CARs was generated as a donor cell to transfer the anti-CD19 CARs onto NK cells via trogocytosis. Anti-CD19 CAR expression was observed in expanded NK cells after these cells were co-cultured for one hour with freeze/thaw-treated donor cells expressing anti-CD19 CARs. Immunofluorescence analysis confirmed the localization of the anti-CD19 CARs on the NK cell surface. Acquisition of anti-CD19 CARs via trogocytosis enhanced NK cell-mediated cytotoxicity against the B-cell acute lymphoblastic leukemia (B-ALL) cell lines and primary B-ALL cells derived from patients. To our knowledge, this is the first report that describes the increased cytotoxicity of NK cells following the acquisition of CARs via trogocytosis. This novel strategy could be a potential valuable therapeutic approach for the treatment of B-cell tumors.

## Introduction

Natural killer (NK) cells have the ability to recognize and eliminate tumor cells, making them ideal candidates for tumor immunotherapy [1], [2]. NK cell activity is regulated by the cumulative effects of multiple activating and inhibitory signals that are transmitted through the receptors on the NK cell surface. We have previously genetically modified *in vitro* expanded NK cells to express DAP10 and the chimeric NKG2D receptor containing the CD3ζ signal domain, which altered the balance between the activating and inhibitory signals of NK cells and enhanced the cytotoxicity against NKG2D ligand-bearing tumors [3]. Further, expression of anti-CD19 chimeric antigen receptors (CARs) containing 41BB and CD3ζ signal domains on NK cells enhanced the activating signals originating from CD19 antigen engagement, leading to cytotoxicity specifically against B-cell leukemia [4].

Trogocytosis is a process in which membrane patches are exchanged between target and immune cells [5]–[7]. When an NK cell interacts with a target cell, an immune synapse, which is strong enough to allow the transfer of small membrane patches from one cell to its partner cell, is formed [8], [9]. Therefore, target cell surface molecules can be found on the surface of NK cells. The chemokine receptor CCR7 has been shown to be transferred from donor cells onto the surface of NK cells via trogocytosis, and this transfer stimulated NK cell migration, leading to enhanced lymph node homing [10], [11]. Similarly, T cells captured NKG2D and NKp46 ligands on tumor cells through trogocytosis and promoted NK cell activity [12].

CD19 is an ideal target antigen for immunotherapy because it is expressed on nearly all leukemia cells in most patients with B-cell acute lymphoblastic leukemia (ALL) and chronic lymphoblastic leukemia (CLL) [13], [14]. T cells expressing anti-CD19 CARs containing 41BB and CD3ζ signaling domains have shown remarkable antileukemic effects, leading to prolonged survival [15], [16]. Autologous T cells transduced with anti-CD19 CARs have been reported to induce complete remission in patients with chronic lymphoblastic leukemia (CLL) and acute lymphoblastic leukemia (ALL) [17]–[20].

In this study, we sought to express anti-CD19 CARs on expanded NK cells to enhance their cytotoxicity against B-ALL cells. Viral vectors have been used to genetically modify expanded NK cells to express CARs [4], [21]. Because of the safety concerns regarding viral integration into the NK cell genome, non-viral mRNA electroporation methods have been developed to modify NK cells and induce NK cell-mediated killing of leukemia cells [22], [23]. Although viral gene transduction and mRNA electroporation are feasible methods, their application is limited because of the high costs and complexity. Therefore, we developed a fast, easy, and low-cost method to modify NK cells via trogocytosis. To the best of our knowledge, this is the first report that describes the use of trogocytosis as a tool to modify NK cells with chimeric antigen receptors to enhance their cytotoxicity against B-cell leukemia cells.

## Materials and Methods

### Cell lines and B-ALL cells from patients

The human B-lineage ALL cell line OP-1 [t(9;22) (q34;q11)/BCR-ABL] was a generous gift from Dario Campana (St. Jude Children's Research Hospital) [24]. The human B-ALL cell lines RS4;11 and SUP-B15 and the non-B leukemia cell line U937 were obtained from American Type Culture Collection (ATCC; Rockville, MD). The K562 cell line was purchased from Bioresource Collection and Research Center (BCRC; Hsinchu, Taiwan). RPMI-1640 (Invitrogen, Carlsbad, CA) supplemented with 10% fetal bovine serum (FBS; Gibco, Carlsbad, CA) and 100 mg/mL penicillin/streptomycin (Invitrogen) was used to maintain K562, OP-1, and RS4;11 cells. The SupB15 cells were maintained in Iscove's Modified Dulbecco's Medium (IMDM; Gibco, Carlsbad, CA).

Following the approval of the protocols and the written informed consent form by the Institutional Review Board of Kaohsiung Veterans General Hospital (Protocol number: VGHKS13-CT6-11), the patients' bone marrow samples were collected strictly adhering to the current ethical principles of the Declaration of Helsinki. Bone marrow samples were collected only after receiving written informed consents from all individuals. The acute B-cell lymphoblastic leukemia cells were separated by centrifugation on a Lymphoprep (Nycomed, Oslo, Norway) density step. The NK cells were thawed and cultured overnight in NK cell culture medium. The cytotoxicity of NK cells against the primary B-cell leukemia cells was assessed immediately after sample collection to avoid massive cell death after the freeze/thaw cycle.

### Human NK cell expansion

Peripheral blood samples were obtained from healthy adult donors. Mononuclear cells collected from the samples by centrifugation on a Lymphoprep (Nycomed, Oslo, Norway) density step were washed twice using medium. To expand the CD56+CD3- NK cells, we co-cultured the peripheral blood mononuclear cells (PBMCs) and the genetically modified K562-mb15-41BBL cell line [4], [25]. PBMCs (3×10^6^) were co-cultured in a 6-well tissue culture plate with 2×10^6^ irradiated or freeze/thaw-treated K562-mb15-41BBL cells in 5 mL of RPMI-1640 medium containing and 10% FBS and 10 IU/mL human IL-2 (eBioscience, San Diego, CA). Once every 2 days, fresh culture medium containing 10% FBS and 20 IU/mL human IL-2 was added to double the volume, and NK cells were split from one well into two wells. After 7 days of co-culture, the T cells were removed using CD3 Dynabeads (Invitrogen, Carlsbad, CA), which yielded cell populations containing >95% CD56+CD3- NK cells. The purified NK cells were stored in liquid nitrogen for further experiments.

For the freeze/thaw cycle, ethanol contained in a 50-mL tube was chilled in a −80°C freezer. Following this, a suspension of K562-mb15-41BBL cells in RPMI medium (5×10^6^/mL) contained in a 1.5-mL tube was rapidly frozen using pre-chilled ethanol for 2 min. The K562-mb15-41BBL cells were then thawed quickly in a water bath at 37°C for co-culturing with PBMCs as described earlier.

### Plasmids, virus production, and gene transduction

The pMSCV-IRES-GFP, pEQ-PAM3(-E), pRDF, and anti-CD19-BB-ζ were generous gifts from Dario Campana (St. Jude Children's Research Hospital) [15]. To generate the RD144-pseudotyped retrovirus, 2.5×10^6^ 293T cells maintained in 10-cm tissue culture dishes for 16 h were transfected with 3.5 µg of cDNA encoding anti-CD19-BB-ζ constructs, 3.5 µg of pEQ-PAM3(-E), and 3 µg of pRDF using fuGENE 6 (Roche, Indianapolis, IN) reagent [4], [15]. The culture supernatant containing the retrovirus was harvested at 48, 72, and 96 h post-transfection. For gene transduction, the supernatant-containing virus particles were filtered and added to RetroNectin (TakaRa, Otsu, Japan)-coated polypropylene tubes. After centrifugation at 1,400×*g* for 10 min, the tubes were incubated at 37°C for 4 h. After additional centrifugation and removal of the supernatant, K562 cells (5×10^4^) were added to the tubes, and the tubes were incubated at 37°C for 24 h. This procedure was repeated for 7 more days. Cells were then maintained in RPMI-1640 supplemented with FBS and antibiotics.

The expression of anti-CD19-BB-ζ on the K562 cell surface was analyzed by flow cytometry on a FACSCalibur instrument using CellQuest software (BD Biosciences, San Jose, CA). Biotin-SP-conjugated AffiniPure goat anti-mouse IgG,F(ab′)2 fragment-specific antibody (Jackson ImmunoResearch 115-065-072) and PE-conjugated streptavidin (Jackson ImmunoResearch 016-110-084) were used for labeling the cells. Single K562-anti-CD19-BB-ζ cells with the highest expression of anti-CD19-BB-ζ were sorted with a FACSAria cell sorter (BD Biosciences, San Jose, CA).

### Trogocytosis and staining

Immediately before trogocytosis, donor or control cells (5×10^6^ cells/mL in culture medium) were rapidly frozen in chilled ethanol (−80°C) for 2 min and then thawed in a water bath maintained at 37°C. Trogocytosis of antiCD19BB-ζ was achieved by co-culturing the NK cells with freeze/thaw-treated K562-antiCD19BBζ cells (donor cells) or K562 cells (control cells) in a 24-well plate as described earlier at an acceptor-to-donor cell ratio of 1∶1. The plate was centrifuged for 2 min to increase cell-cell contact and was then incubated at 37°C. After trogocytosis, the cells were gently pipetted to disrupt cell-cell interaction of the immune synapses. The NK cells were separated from the donor or control cells by density gradient centrifugation on Lymphoprep (Nycomed, Oslo, Norway) at 400×*g* for 20 min and were cultured in NK cell medium. For staining, the cells were fixed with 1% para in PBS for 15 min at room temperature. After washing, the cells were stained with biotin-SP-conjugated AffiniPure goat anti-mouse IgG,F(ab′)2 fragment-specific antibody (Jackson ImmunoResearch 115-065-072), followed by PE-conjugated streptavidin (Jackson ImmunoResearch 016-110-084) and CD56-FITC. The percentage of the NK cells that acquired anti-CD19-BB-ζ CARs through trogocytosis was determined by flow cytometry.

### Immunofluorescence analysis

The NK cells co-cultured with donor cells were fixed using 4% paraformaldehyde in PBS, permeabilized with 0.4% Triton X-100, and blocked with 2% goat serum for 15 min. The cells were then incubated with biotin-SP-conjugated AffiniPure goat anti-mouse IgG,F(ab′)2 fragment-specific antibody (Jackson ImmunoResearch 115-065-072) at 4°C overnight. After washing, the cells were incubated with Alexa Fluor 568-conjugated streptavidin (Invitrogen, Carlsbad, CA) and CD56-FITC antibodies (BD Biosciences, San Jose, CA). DAPI was used for staining the nucleus. The localization of fluorescently labeled protein was visualized using a fluorescence microscope (Carl Ziess, Jena, Germany).

### Cytotoxicity assay

The target cells were suspended in RPMI-1640 containing 10% FBS, labeled with calcein AM (BD Biosciences, San Jose, CA), and plated onto 96-well flat-bottom plates (Costar, Corning, NY). The NK cells, suspended in RPMI-1640 containing 10% FBS, were then added at various E:T ratios and co-cultured with the target cells for 4 h. Following this, the cells were stained with propidium iodide (Sigma-Aldrich, St. Louis, MO), and the cytotoxicity was assessed by flow cytometry on a FACSCalibur (Becton Dickinson) instrument enumerating the number of viable target cells (calcein AM-positive, propidium-iodide negative, and light scattering properties of viable cells) [3], [26].

### Degranulation assays

NK cells (1×10^5^) were plated in each well of a 96-well flat-bottom plate and incubated with RS4;11 cells at an E:T ratio of 1∶4. A phycoerythrin-conjugated anti-human CD107a antibody (BD Biosciences, San Jose, CA) was added at the beginning of the cultures. After 1 h of incubation, GolgiStop (0.15 µL; BD Biosciences, San Jose, CA) was added. The cells were then stained with fluorescein isothiocyanate-conjugated anti-human CD56 antibody (eBiosciences, San Diego, CA) and were analyzed by flow cytometry.

## Results

### Freeze/thaw treated K562-mbIL15-41BBL cells can be used in NK cell expansion

**Figure 1:**
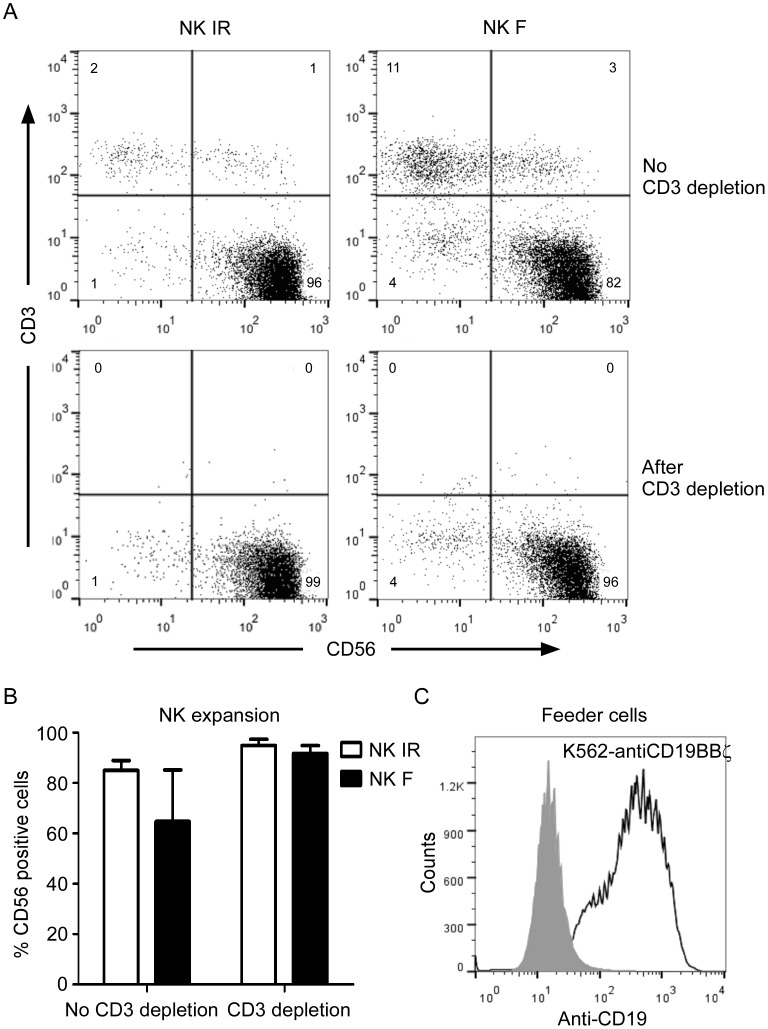
Immunophenotypic features of expanded NK cells (acceptor cells) and K562-antiCD19BBζ cells (feeder cells). A. Expression of CD56 and CD3 on peripheral blood mononuclear cells from a healthy donor was examined after 1 week (top row) of co-culture with irradiated (IR, left column) or freeze/thaw-treated (F, right column) K562-mb15-41BBL cells at a low dose (10 U/mL) of IL-2. The T cells were removed using CD3 Dynabeads, generating cell populations comprising >95% CD56+CD3- NK cells (bottom row). B. Percentage of CD56-positive cells within NK cells expanded by co-culturing with irradiated (IR) or freeze/thaw-treated (F) K562-mb15-41BBL cells prior to and after CD3 depletion on day 7. The data are presented as the mean of values obtained using 3 unrelated NK donors. Error bars represent the SD. C. Histogram illustrating the anti-CD19 expression on K562 cells (control, shaded histogram) and K562-antiCD19BBζ cells (feeder cells, open histogram).

The NK cells were expanded from PBMCs by co-culturing with irradiated (Figure 1A, left panels) or freeze/thaw-treated (Figure 1A, right panels) K562-mb15-41BBL cells. The freeze/thaw cycle compromised the membrane integrity of K562-mb15-41BBL cells, which allowed trypan blue staining, but intact cell morphology was maintained. After 7 days of expansion, the PBMCs co-cultured with irradiated K562-mbIL15-41BBL cells produced 95.8% CD56+CD3- NK cells (Figure 1A, left upper panel), whereas K562-mbIL15-41BBL cells subjected to one freeze/thaw cycle yielded 81.8% CD56+CD3- cells (Figure 1A, right upper panel). After CD3 depletion, the percentages of CD56+CD3- NK cells were 98.7% from PBMCs co-cultured with irradiated K562-mbIL15-41BBL cells and 95.5% using a freeze/thawed procedure (Figure 1A, lower panels). The relatively poor expansion of NK cells in co-cultures of PMBCs and freeze/thaw-treated K562-mbIL15-41BBL cells was likely due to the reduced stimulation from mbIL15 and 41BB ligands as a result of freeze/thaw-induced cell damage and lysis.

By day 7 in cultures, approximately 85% (n = 3; Figure 1B, NK IR) of the NK cells expanded from PBMCs co-cultured with irradiated K562-mb15-41BBL cells were CD56-positive, and this number increased to 95% following CD3 depletion. These results were comparable to those of NK cells expanded by co-culturing with freeze/thaw-treated K562-mb15-41BBL cells (Figure 1B, NK F; 92% CD56-positive cells following CD3 depletion). These results indicated that both freeze/thaw-treated and irradiated K562-mbIL15-41BBL cells can be used for NK cell expansion.

To generate donor cells (K562-antiCD19BBζ cells) for trogocytosis, K562 cells were transduced with anti-CD19-BB-ζ chimeric antigen receptors (CARs). After single cell sorting, we chose the clone stably expressing high levels of anti-CD19 CARs. The mean fluorescence intensity (MFI) of the K562-antiCD19BBζ cells was 301, which was substantially higher than that (12) of control K562 cells (Figure 1C).

### Expanded NK cells acquired anti-CD19 CARs from K562-based donor cells via trogocytosis

**Figure 2:**
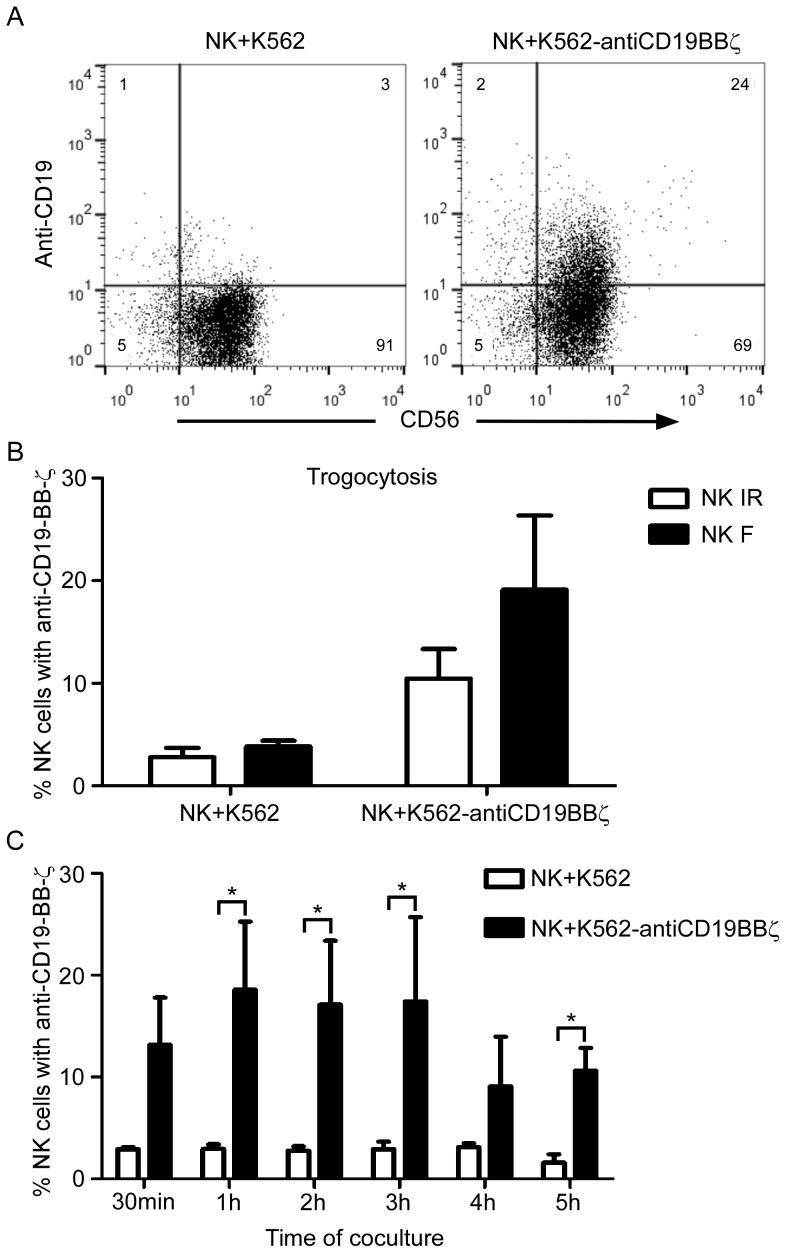
Acquisition of anti-CD19 chimeric antigen receptors (CARs) by NK cells from donor cells via trogocytosis. A. Flow cytometry dot plots illustrating the uptake of anti-CD19 CARs by NK cells via trogocytosis. NK cells co-cultured with K562 cells (control) or K562-antiCD19BBζ cells were stained with an anti-CD56-FITC antibody and a biotin-SP-conjugated AffiniPure goat anti-mouse IgG,F(ab′)2 fragment specific antibody, followed by PE-conjugated streptavidin. B. Uptake of anti-CD19 CARs by NK cells expanded by co-culturing with irradiated (IR) or freeze/thaw-treated (F) K562-mb15-41BBL cells. The data are presented as the mean ± SD of values obtained using three unrelated NK donors. C. Kinetics of anti-CD19 CAR uptake by NK cells from K562-antiCD19BBζ cells (black bars) and control K562 cells (white bars). The uptake of anti-CD19 CARs by NK cells was analyzed after co-culturing with feeder cells for the indicated time and was compared with that of NK cells co-cultured with control K562 cells. The data are presented as the mean ± SD of values obtained using 3 unrelated NK cell donors. *: significant increase compared with control cells (p<0.05; two-tailed paired Student's *t*-tests).

To examine whether the NK cells were able to acquire anti-CD19 CARs from donor cells via trogocytosis, we cultured NK cells with live or freeze/thaw-treated donor K562-antiCD19BBζ cells. FACS analysis of NK cells co-cultured with live donor cells revealed that 47.0±16.4% (n = 3, ± s.d.) of the NK cells acquired anti-CD19 CARs (data not shown), and 24.1% of NK cells acquired anti-CD19 CARs from freeze/thawed donor K562-antiCD19BBζ cells (Figure 2A). Although a higher efficiency of trogocytosis was observed in co-cultures with live donor cells, separation of NK cells from live donor cells is challenging. In contrast, donor cells subjected to a freeze/thaw cycle could facilitate the separation of NK cells from nonviable donor cells using Ficoll-Paque centrifugation. Therefore, the NK cells co-cultured with freeze/thaw-treated K562-antiCD19BBζ cells were used for subsequent experiments.

Using these two types of expanded NK cells (Figure 1A, NK IR and NK F), we evaluated the uptake of anti-CD19 CARs by NK cells from donor K562-antiCD19BBζ cells. We found that approximately 19% (n = 3) of the NK F cells expressed anti-CD19 CARs, whereas only 11% (n = 3) of the NK IR cells expressed anti-CD19 CARs (Figure 2B). Therefore, we used NK cells expanded with freeze/thaw-treated K562-mb15-41BBL cells (NK F cells) in subsequent trogocytosis experiments.

We assessed the optimal duration for the co-culture of NK cells with donor cells. After 1 h of co-culture, approximately 18.6% of the NK cells were anti-CD19-positive (Figure 2C). The uptake of anti-CD19 CARs peaked at 1 h and decreased after 4–5 h of co-culture with K562-antiCD19BBζ cells. These results suggested that the efficiency of NK cell trogocytosis peaked at 1 h of co-culture. Therefore, we used 1 h as the standard duration of co-culture in subsequent experiments.

### Immunofluorescence analysis of trogocytosis

**Figure 3:**
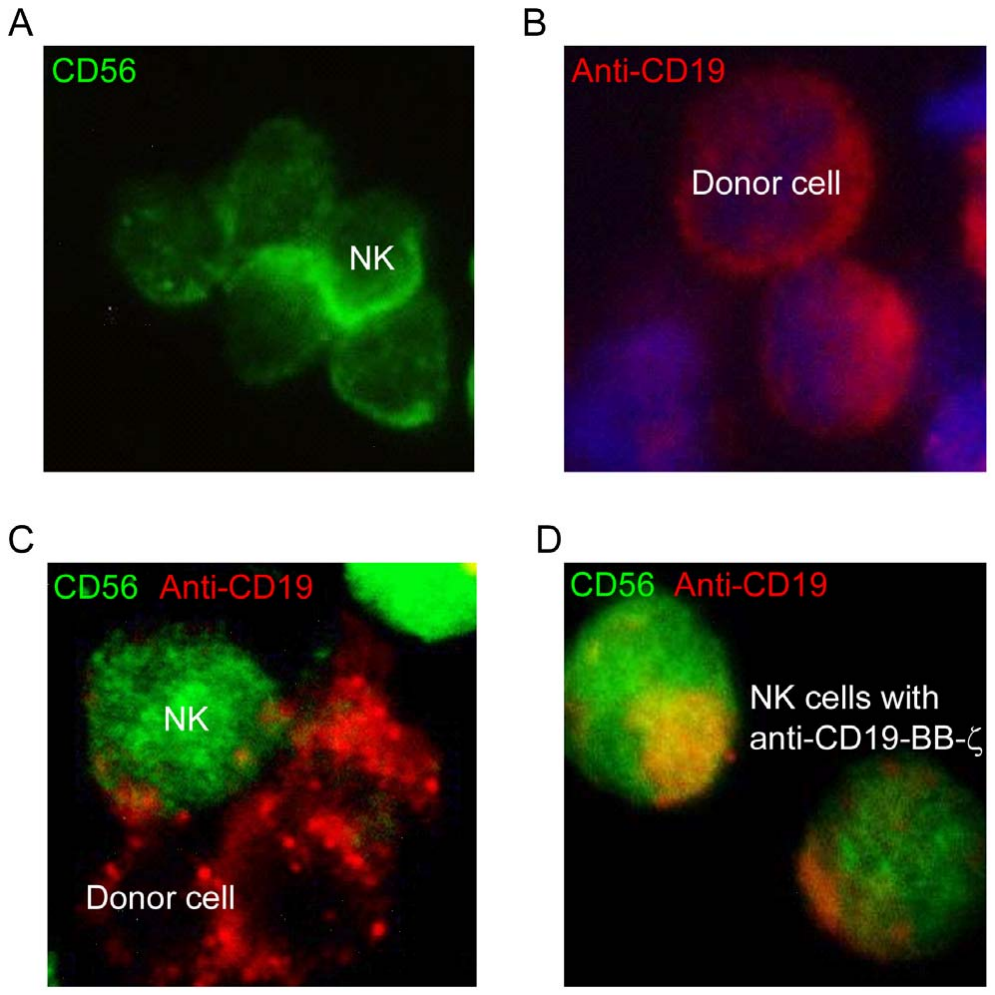
Immunofluorescence analysis for trogocytosis. A. NK cells stained with anti-CD56-FITC antibody. B. K562-antiCD19BBζ cells were stained with a biotin-SP-conjugated AffiniPure goat anti-mouse IgG,F(ab′)2 fragment specific antibody, followed by Alexa Fluor 568-conjugated streptavidin. The nucleus was stained with DAPI (blue). C. NK cells co-cultured with K562-antiCD19BBζ cells were stained for CD56 and anti-CD19 CARs, as previously described. D. Acquisition of anti-CD19-BB-ζ by NK cells via trogocytosis was observed.

To verify that trogocytosis was the mechanism of uptake of anti-CD19 CARs by NK cells, we examined the interaction between NK cells and donor cells during trogocytosis using immunofluorescence microscopy. The NK cells stained positively for CD56 (Figure 3A, green), and the donor cells expressed anti-CD19 CARs (Figure 3B, red). Because the NK cells interacted with donor cells and formed immune synapses over a period of 15 min, these cells were imaged 15 min after co-culture initiation. During trogocytosis, small patches of acquired anti-CD19 CARs were observed on the surface of NK cells (Figure 3C). The NK cells continued to express anti-CD19 CARs (Figure 3D, yellow) on their surfaces after detaching from the donor cells following pipetting and Ficoll-Paque separation.

### Acquiring anti-CD19 CARs via trogocytosis enhanced NK cell degranulation and cytotoxicity against B-ALL cell lines

**Figure 4:**
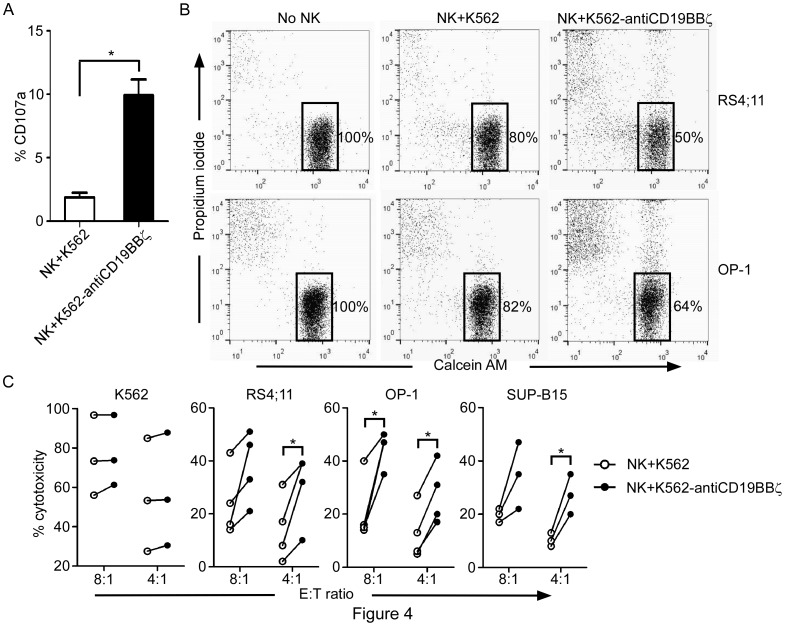
Degranulation and cytotoxicity assays. A. Incubation of NK cells with RS4;11 cells induced degranulation, which was significantly higher in NK cells co-cultured with K562-antiCD19BBζ cells than in NK cells cultured with control K562 cells. Percentages of CD56-positive cells from 3 donors expressing CD107a after a 4-h RS4;11 stimulation are shown. The data are presented as the mean ± SD of values obtained using 3 unrelated NK cell donors. B. Flow cytometric dot plots illustrating the assay used to measure cell killing. Results for RS4;11 and OP-1 cell lines are shown. Tumor cells were either cultured alone (left panel), with NK cells previously co-cultured with K562 cells (middle panel), or with NK cells previously co-cultured with K562-antiCD19BBζ cells (right panel). Residual viable target cells, which were defined as calcein AM-positive and propidium iodide (PI)-negative, are shown in the bottom right corner of each panel, and the percentages of viable cells are shown. C. Cytotoxicity of the non-B leukemia cell line (K562) and B-ALL cell lines (RS4;11, OP1, and SUP-B15) after 4-h co-culture with NK cells previously co-cultured with K562 cells (white circles) and K562-antiCD19BBζ cells (black circles) at the indicated E:T ratios. The equation [(tumor co-culture with NK cells)/(tumor alone)]×100%, represents the quantitative percentage of viable tumor cells. The cytotoxicity was calculated according the following equation: 100% – quantitative percentage of viable tumor cells. Each symbol corresponds to the mean of three values. *: significant increase compared with control cells (p<0.05; two-tailed paired Student's *t*-tests).

To assess the degranulation of NK cells after stimulating with a B-ALL cell line, NK cells co-cultured with freeze/thaw-treated donor K562-antiCD19BBζ or K562 (control) cells were incubated with RS4;11 cells to induce degranulation. CD107a staining was performed to detect degranulation. The percentage of CD107a-positive NK cells co-cultured with donor K562-antiCD19BBζ cells (9.9±1.2%, n = 3) was significantly higher (*p*<0.05) than that of CD107a-positive NK cells co-cultured with control cells (1.9±0.6%, n = 3) (Figure 4A).

To examine whether the gain of anti-CD19 CARs via trogocytosis improved the cytotoxicity of NK cells against B-ALL cells, three B-ALL cell lines (RS4;11, OP-1, and SUP-B15) were targeted. We conducted 4-h cytotoxicity assays with NK cells expanded from three donors at effector:target (E:T) ratios of 4∶1 and 8∶1. As shown in Figure 4B and 4C, the gain of anti-CD19 CARs via trogocytosis significantly increased the cytotoxicity of NK cells against RS4;11, OP-1, and SUP-B15 cells at a 4∶1 E:T ratio. Similarly, gains of cytotoxicity were also observed against the three B-ALL cell lines at an 8∶1 E:T ratio. In contrast, there was no increase in cytotoxicity against the non-B cell lines, K562 cells (Figure 4C) and U937 cells (data not shown).

### Gain of anti-CD19 CARs via trogocytosis increased NK cytotoxicity against primary B-ALL cells from patients

**Figure 5:**
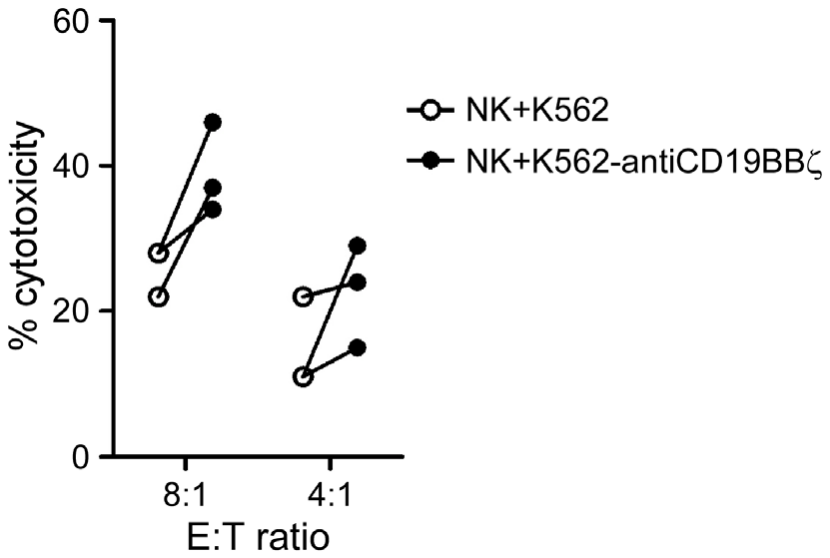
NK cells that acquired anti-CD19 CARs were more cytotoxic against patient-derived primary B-ALL cells. Cytotoxicity against B-ALL cells after 4-h co-culture with NK cells previously co-cultured with K562 cells (white circles) and K562-antiCD19BBζ cells (black circles) at the indicated E:T ratios. Each symbol corresponds to the mean of three values.

To determine whether the acquisition of anti-CD19 CARs via trogocytosis improved the cytotoxicity of NK cells against the patient-derived primary B-ALL cells, three samples of bone marrow from B-ALL patients were tested. Compared to the mock NK cells, the NK cells that acquired anti-CD19 CARs consistently showed enhanced cytotoxicity against primary B-ALL cells (Figure 5). The p-value was 0.069 (n = 3) at an 8∶1 E:T ratio.

## Discussion

Because of the safety concerns regarding the use of viral transduction methods, we endeavored to improve the cytotoxicity of adoptively transferred NK cells against B-cell leukemia cells using a simple co-culture method. For NK cell therapy, the clinical protocol required up to 5× 10^7^ NK cells/kg and CD3 depletion before transfusing into patients, indicating that approximately 10×10^9^ NK cells are needed for the treatment of hematological malignancies [27]. If the MSCV retroviral methods were to be used to modify NK cells, a large quantity of viral supernatant would be required to transduce 10×10^9^ NK cells [4]. Additionally, there is a safety concern associated with viral integration into the NK cell genome. Further, the application of viral transduction is also very limited because of the complexity of the procedures and high costs. In this study, we evaluated the usefulness of trogocytosis as a relatively simple non-viral method that can be readily scaled up to modify large numbers of NK cells with a single K562-based donor cell line.

The K562 cell line is a typical NK cell target because it lacks major histocompatibility complex class I expression, which triggers inhibitory signals to abolish NK cell activation. Additionally, K562 cells have been genetically modified to express 41BBL and the membrane-bound IL15 or IL21, which allowed the establishment of co-culture methods for the *in vitro* expansion of NK cells [25], [28]. Here, we showed that K562 cells were able to donate anti-CD19 CARs to expanded NK cells in co-cultures via trogocytosis. The rapid expression of anti-CD19 CAR on the NK cell surface when co-cultured with donor K562-antiCD19BBζ cells and the lack of expression in NK cells co-cultured with control K562 cells was suggestive of an acquisition from donor cells. This was confirmed by the positive anti-CD19 staining of the NK cell surface (Figure 2A, Figure 4). Previous studies have shown that trogocytosis occurs during cell-cell interactions between target cells and stimulated NK cells [9]–[11], [29], [30].

Although the extent of trogocytosis was greater when NK cells were co-cultured with live donor cells (47%, data not shown) than when cultured with nonviable donor cells (19%), the ease of separating NK cells from nonviable donor cells using Ficoll-Paque centrifugation prompted us to use the latter method. The uptake of CCR7 by NK cells from nonviable donor cells subjected to one freeze/thaw cycle was also lower (50%) than that from live donor cells (80%) [10]. Further, the anti-CD19 CARs acquired by NK cells were rapidly lost, and only less than 20% of the acquired anti-CD19 CARs remained on the NK cell surface after 2 h (data not shown). CCR7 uptake has been reported to decline to baseline levels by 72 h [10]. The rapid loss of the acquired anti-CD19 CARs observed in our study might be due to the degradation of the low amount of anti-CD19 CARs acquired. Fas signaling was reported to promote trogocytosis in T cells [31]. Further studies are needed to improve anti-CD19 CAR uptake and enhance its stability on the NK cell surface.

The CAR, anti-CD19-BB-ζ, comprising an anti-CD19 single-chain variable fragment (scFv), a 41BB signaling domain, and a CD3ζ signaling domain, was transduced into the NK cell genome, inducing powerful anti-leukemic effects [4]. Additionally, expanded NK cells electroporated with anti-CD19-BB-ζ mRNA also exerted significantly higher cytotoxicity against B-cell malignancies than mock NK cells [23]. In this study, we showed enhanced cytotoxicity of NK cells following the acquisition of anti-CD19-BB-ζ protein molecules via trogocytosis.

A reduction in NK cell cytotoxicity was observed after the intercellular transfer of NK Group 2 member D (NKG2D) and MHC class I chain-related molecule (MIC) B proteins at the NK cell immune synapse [32]. The amount of NKG2D, a key activating receptor on NK cell surface, is reduced after trogocytosis because the transfer of NK cell-derived NKG2D to target cells and internalization of NKG2D contribute to receptor down-modulation after interaction, leading to impaired NK cell cytotoxicity [7], [32]. In addition, activated NK cells acquired HLA-G, an immunosuppressive molecule, from tumor cells via trogocytosis, leading to impaired cytotoxicity [9]. On the contrary, our study showed enhanced NK cytotoxicity because of expanded NK cells acquiring anti-CD19-BB-ζ from feeder cells via trogocytosis.

To our knowledge, this is the first report that describes the increased cytotoxicity of NK cells following the acquisition of anti-CD19 CARs from donor cells via trogocytosis. Our findings could potentially be extended to develop safer and effective therapeutic strategies for treating B-cell tumors. Our model, which used anti-CD19 CAR for targeting a B-cell tumor, could also be relevant to other tumor types that are targeted by the tumor-directed chimeric antigen receptors [33]. Therefore, further studies are warranted to examine the utility of our method for treating diverse tumor types *in vivo*.
